# Transatlantic spread of highly pathogenic avian influenza H5N1 by wild birds from Europe to North America in 2021

**DOI:** 10.1038/s41598-022-13447-z

**Published:** 2022-07-11

**Authors:** V. Caliendo, N. S. Lewis, A. Pohlmann, S. R. Baillie, A. C. Banyard, M. Beer, I. H. Brown, R. A. M. Fouchier, R. D. E. Hansen, T. K. Lameris, A. S. Lang, S. Laurendeau, O. Lung, G. Robertson, H. van der Jeugd, T. N. Alkie, K. Thorup, M. L. van Toor, J. Waldenström, C. Yason, T. Kuiken, Y. Berhane

**Affiliations:** 1 Department of Viroscience, Erasmus University Medical Center, Rotterdam, The Netherlands; 2 Department of Pathobiology and Population Sciences, Royal Veterinary College, Hatfield, UK; 3 Institute of Diagnostic Virology, Friedrich-Loeffler-Institut, Greifswald-Insel Riems, Germany; 4 Animal and Plant Health Agency, Addlestone, UK; 5 Vogeltrekstation-Netherlands Institute of Ecology NIOO-KNAW, Wageningen, The Netherlands; 6 Department of Biology, Memorial University of Newfoundland, St. John’s, Canada; 7 Canadian Food Inspection Agency, Winnipeg, Canada; 8 Environment and Climate Change Canada, Mount Pearl, Canada; 9 Linnaeus University, Kalmar, Sweden; 10 Atlantic Veterinary College, University of Prince Edward Island, Charlottetown, Canada; 11 British Trust for Ornithology, Norfolk, UK; 12 Natural History Museum of Denmark, University of Copenhagen, Copenhagen, Denmark; 13 Center for Macroecology, Evolution and Climate, Globe Institute, University of Copenhagen, Copenhagen, Denmark; 14 Department of Coastal Systems, Royal Netherlands Institute for Sea Research, Den Burg, The Netherlands; 15 European Union for Bird Ringing C/O British Trust for Ornithology, Norfolk, UK

**Keywords:** Microbiology, Ecology, Diseases

## Abstract

Highly pathogenic avian influenza (HPAI) viruses of the A/Goose/Guangdong/1/1996 lineage (GsGd), which threaten the health of poultry, wildlife and humans, are spreading across Asia, Europe, Africa and North America but are currently absent from South America and Oceania. In December 2021, H5N1 HPAI viruses were detected in poultry and a free-living gull in St. John’s, Newfoundland and Labrador, Canada. Our phylogenetic analysis showed that these viruses were most closely related to HPAI GsGd viruses circulating in northwestern Europe in spring 2021. Our analysis of wild bird migration suggested that these viruses may have been carried across the Atlantic via Iceland, Greenland/Arctic or pelagic routes. The here documented incursion of HPAI GsGd viruses into North America raises concern for further virus spread across the Americas by wild bird migration.

## Introduction

The A/Goose/Guangdong/1/96 (GsGd) lineage of highly pathogenic avian influenza (HPAI) H5 virus first emerged in poultry in Southeast Asia more than 25 years ago. During the first decade of circulation of this lineage, the hemagglutinin (H) genes diversified into multiple genetic clades. GsGd viruses of clade 2.3.4.4 started to dominate outbreaks globally from 2014 onwards with clade 2.3.4.4b currently emerging as a particularly fit virus. The GsGd lineage, and particularly clade 2.3.4.4b, is expanding both its geographical spread and its host range^1–4^. Therefore, this lineage of HPAI H5 virus is an increasing threat to the health of poultry, wildlife, and humans worldwide, as well as a growing economic problem for the global poultry sector^3,5^.

In recent years, HPAI GsGd H5 outbreaks have frequently occurred in Europe^3–8^. For the first time in 2005, the virus spread from Asia to Russia, western Europe, Africa and the Middle East, causing high mortality in wild birds and poultry^8^. This spread was a result of unprecedented long-distance transport of HPAI virus, in which wild migratory ducks, geese and swans were implicated. The last wild bird case for clade 2.2 and derivatives in Europe was detected in Germany in January 2009^6^. Another European incursion of a clade 2.3.2.1 virus occurred in 2009 but was not detected further west than Romania and Bulgaria, after being detected in Qinghai in China and Tyva in Russia^7^. In 2014/2015 a new intercontinental outbreak of HPAI H5 virus occurred, and since then HPAI H5 viruses have repeatedly caused large outbreaks in wild birds and poultry in Europe^5,6^. In addition, there are also growing concerns about the zoonotic risks, and in December 2021, the European Centre for Disease Prevention and Control raised the risk level for virus transmission to occupationally exposed people from ‘low’ to ‘low/moderate’^5,9^.

In December 2021, there was a die-off of domestic birds on an exhibition farm in St. John’s, a city on the Avalon Peninsula of the island of Newfoundland, on the Atlantic coast of Canada. The cause was diagnosed as HPAI H5N1. This was the first report of HPAI H5 in the Americas since June 2015, when the virus spread with wild birds across the Bering Strait to the Pacific coasts of Canada and the USA via the Pacific Flyway, one of the main avian migration routes^10^. Genetic analysis as reported in the OIE report showed that the hemagglutinin (H) gene corresponded to Eurasian HPAI viruses circulating in spring 2021^11^. This implied that the virus had been carried across the Atlantic, a route that has been recorded before for LPAI viruses but not for any HPAI virus^12,13^. Therefore, the goal of this study was 1) to investigate in detail whether the HPAI cases in Newfoundland were linked to the recent (2020/2021) or currently ongoing (2021/2022) HPAI outbreaks in Europe, and 2) to indicate the most likely scenario by which the virus crossed the Atlantic with migratory birds.

## Results

### Epidemiological description of exhibition farm outbreak

The index farm where highly pathogenic avian influenza (HPAI) H5N1 virus in captive birds occurred was an exhibition farm in St. John’s, Province of Newfoundland and Labrador, Canada. The farm housed 409 birds of different species, namely chickens, guineafowl, peafowl, emus, domestic ducks, domestic geese, and domestic turkeys. On 9th December 2021, the farm owner first noticed mortality. On 13th December, the farm owner reported the increased mortality to a local veterinarian. Autopsies on four chickens showed swollen heads and cutaneous haemorrhages. Oropharyngeal and cloacal swabs from these chickens tested positive for H5 avian influenza virus at the Atlantic Veterinary College, University of Prince Edward Island, and the Canadian Food Inspection Agency (CFIA) was notified. On 16th December, by which time 306 birds (mostly chickens, turkeys and guineafowl) had died, staff of the CFIA collected tissue samples from dead chickens, as well as oropharyngeal and cloacal swabs and sera from different species of captive birds still present (Table 1), after which all remaining captive birds were culled. All oropharyngeal and cloacal swabs tested positive for HPAI virus of the subtype H5N1 by real-time RT-PCR, and all sera tested positive for influenza nucleoprotein antibodies by ELISA. On 20th December, the CFIA confirmed the diagnosis of HPAI of the subtype H5N1.

**Table 1:** List of samples for virological and serological analysis collected by CFIA on 17 December 2021 from different species of captive birds still present at the farm.

Bird type	Number on site	Number of oropharyngeal swabs	Number of cloacal swabs	Number of sera
Turkey (*Meleagris* *gallopavo*)	4	4	4	4
Silkie chicken (*Gallus* *gallus*)	8	8	8	0
Emus (*Dromaius* *novaehollandiae*)	2	2	0	0
Peafowl (*Pavo* *cristatus*)	5	5	5	0
Domestic goose (*Anser* *anser*)	14	0	14	5
Domestic duck (*Anas* *platyrhynchos* *domesticus*)	20	0	20	7
Guineafowl (*Numida* *meleagris*)	0	0	0	0
Chicken-other (*Gallus* *gallus*)	4	4	4	1
Total	57	23	55	17

Wild birds were frequently observed co-mingling with the captive population. Captive birds except emus were allowed to move freely in and out of the open pens in which they were housed. At an on-site pond, domestic ducks were reported to mingle with free-living mallards (scientific names of wild birds in Table 2), and a snowy egret had been observed around 2nd to 6th December. Other wild birds reported on the farm were common starlings, feral pigeons, and unspecified gulls.

**Table 2:** Common and scientific species names of the birds mentioned in the text.

Common name	Scientific name
Atlantic puffin	*Fratercula* *arctica*
Black-legged kittiwake	*Rissa* *tridactyla*
Barnacle goose	*Branta* *leucopsis*
Black-headed gull	*Chroicocephalus* *ridibundus*
Brunnich’s guillemot	*Uria* *lomvia*
Common eider	*Somateria* *mollissima*
Common starling	*Sturnus* *vulgaris*
Eurasian teal	*Anas* *crecca*
Eurasian wigeon	*Mareca* *penelope*
Feral pigeon	*Columba* *livia* *domestica*
Great black-backed gull	*Larus* *marinus*
Great skua	*Stercorarius* *skua*
Greater white-fronted goose	*Anser* *albifrons*
Greylag goose	*Anser* *anser*
Lesser black-backed gull	*Larus* *fuscus*
Light-bellied brent goose	*Branta* *bernicla*
Mallard	*Anas* *platyrhynchos*
Northern fulmar	*Fulmarus* *glacialis*
Northern pintail	*Anas* *acuta*
Pink-footed goose	*Anser* *brachyrhynchus*
Purple sandpiper	*Calidris* *maritima*
Red knot	*Calidris* *canutus*
Snow goose	*Anser* *caerulescens*
Snowy egret	*Egretta* *thula*
Tufted duck	*Aythya* *fuligula*
Whooper swan	*Cygnus* *cygnus*

Retrospectively, HPAI H5N1 virus also was identified in tissues of a great black-backed gull found at a nearby pond in St. John’s. The gull had been found ill on 26th November 2021 and taken to a local wildlife rehabilitation centre, where it died the following day.

### Phylogenetic analysis

**Figure 1:**
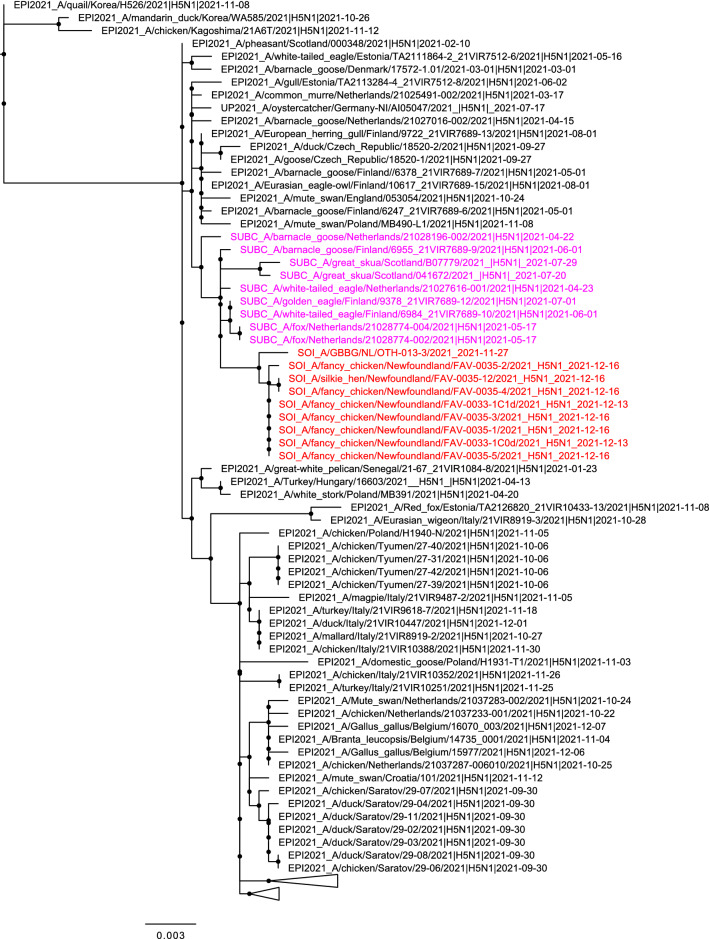
Maximum likelihood phylogenetic tree of the H5 HA gene. Relationships among the European 2021 H5 2.3.4.4b HPAI strains (magenta) and the Newfoundland wild bird and outbreak strains (red) are shown. The tree is rooted by the outgroup and nodes placed in descending order. Clades are collapsed for clarity.

**Figure 2:**
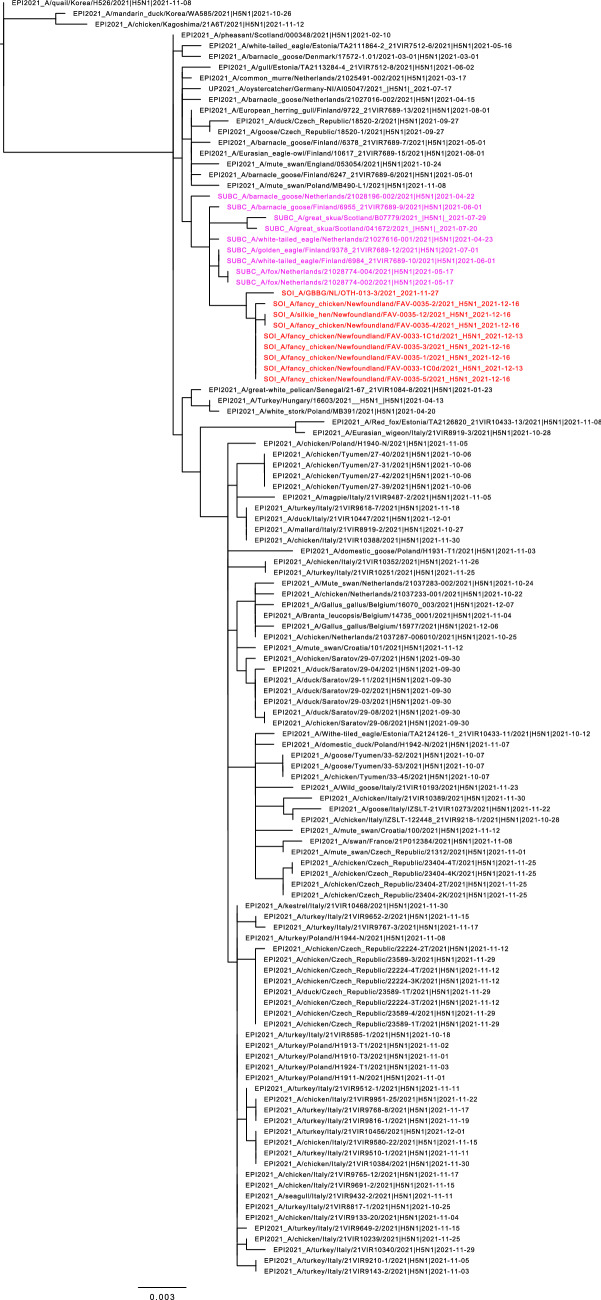

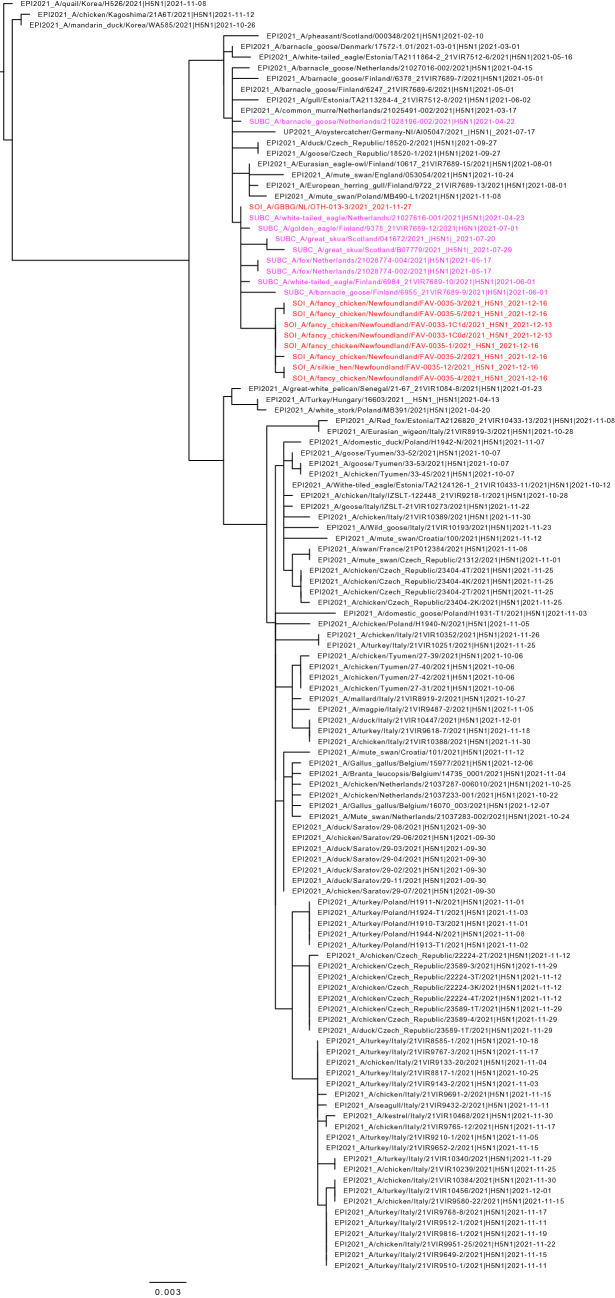

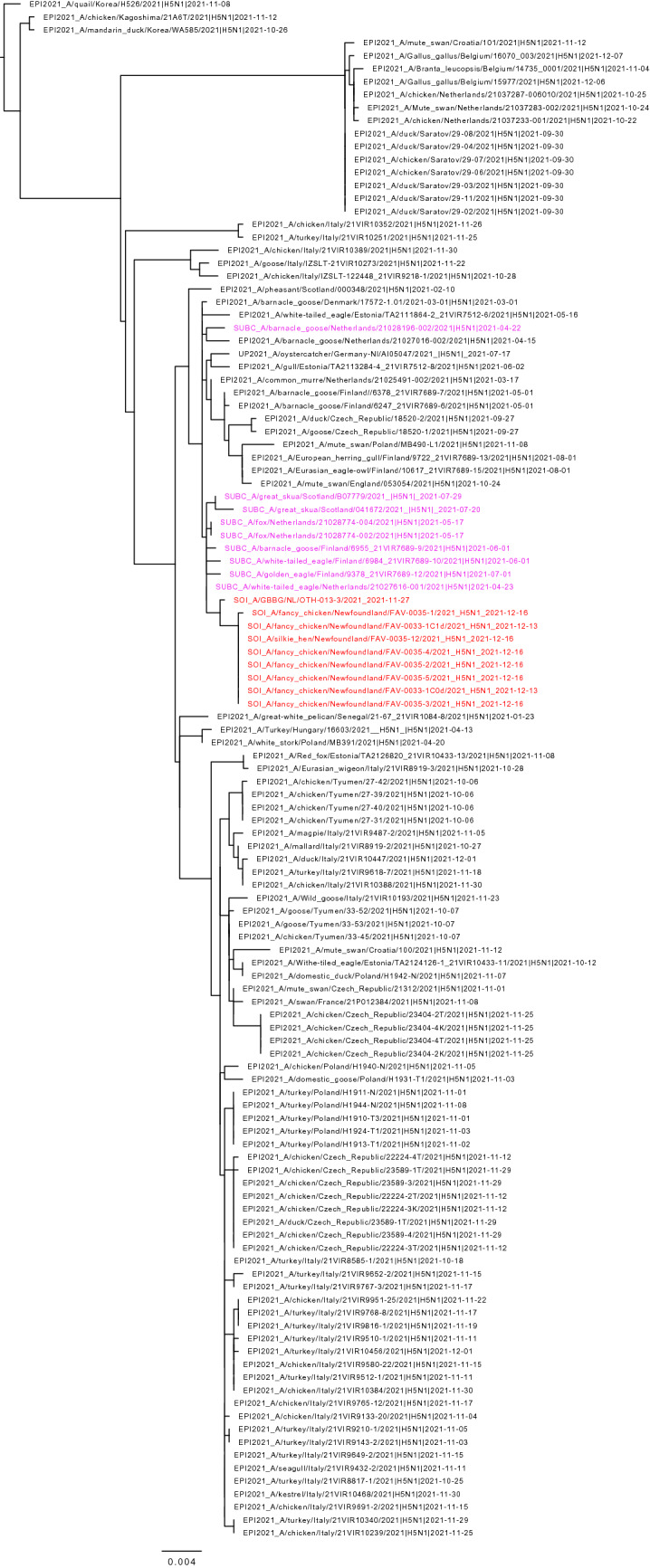

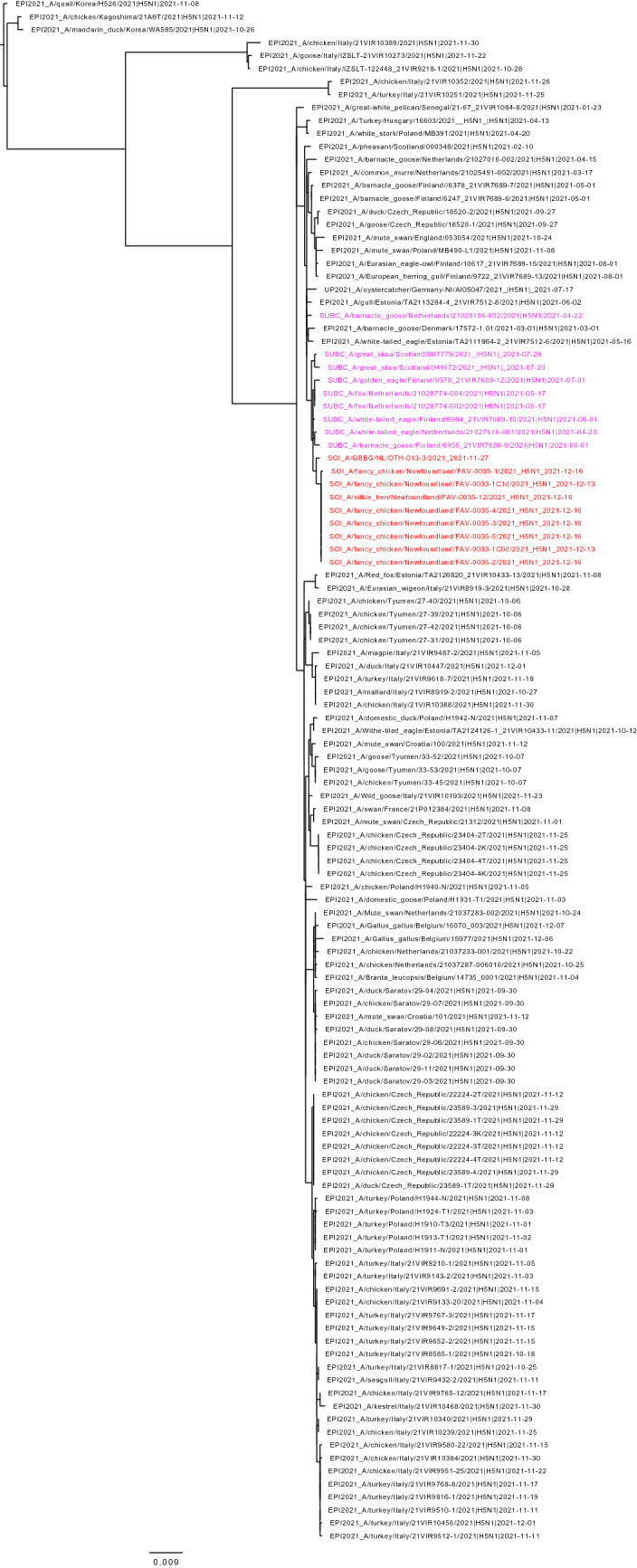

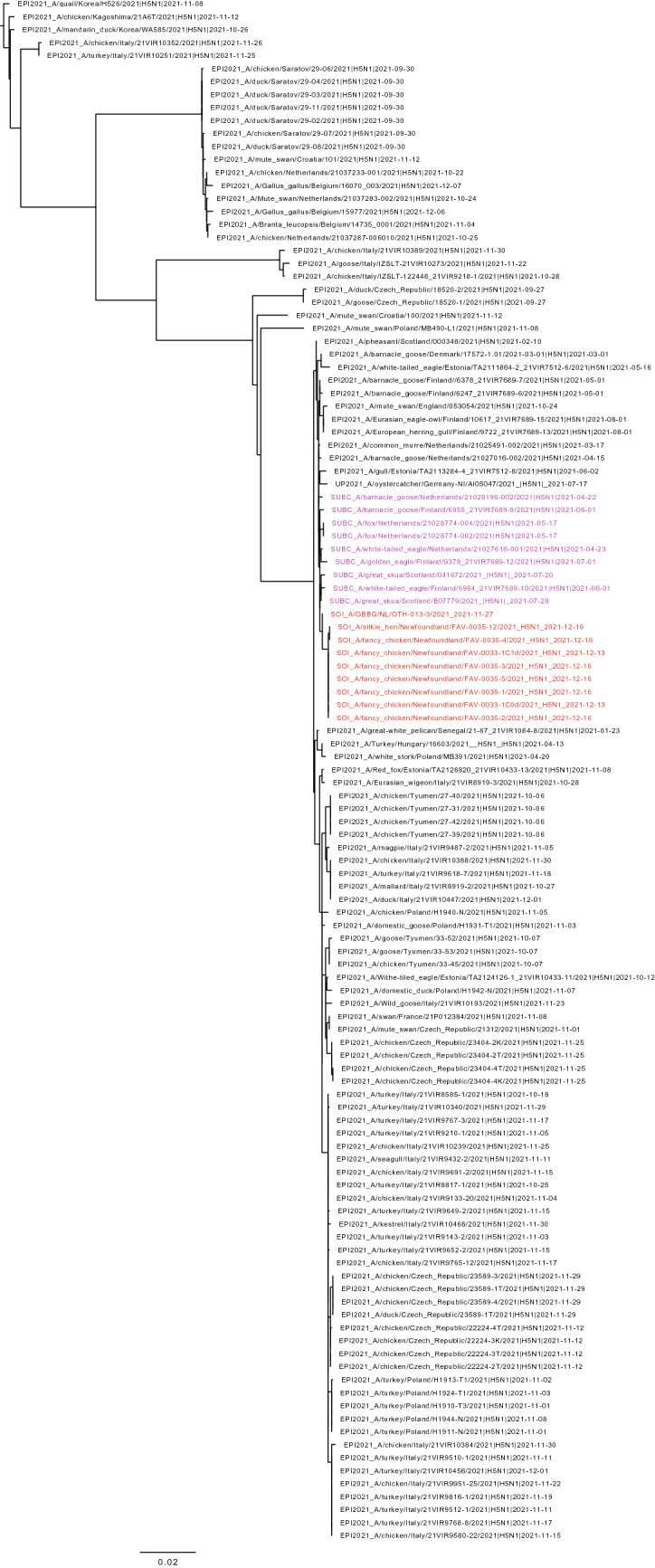

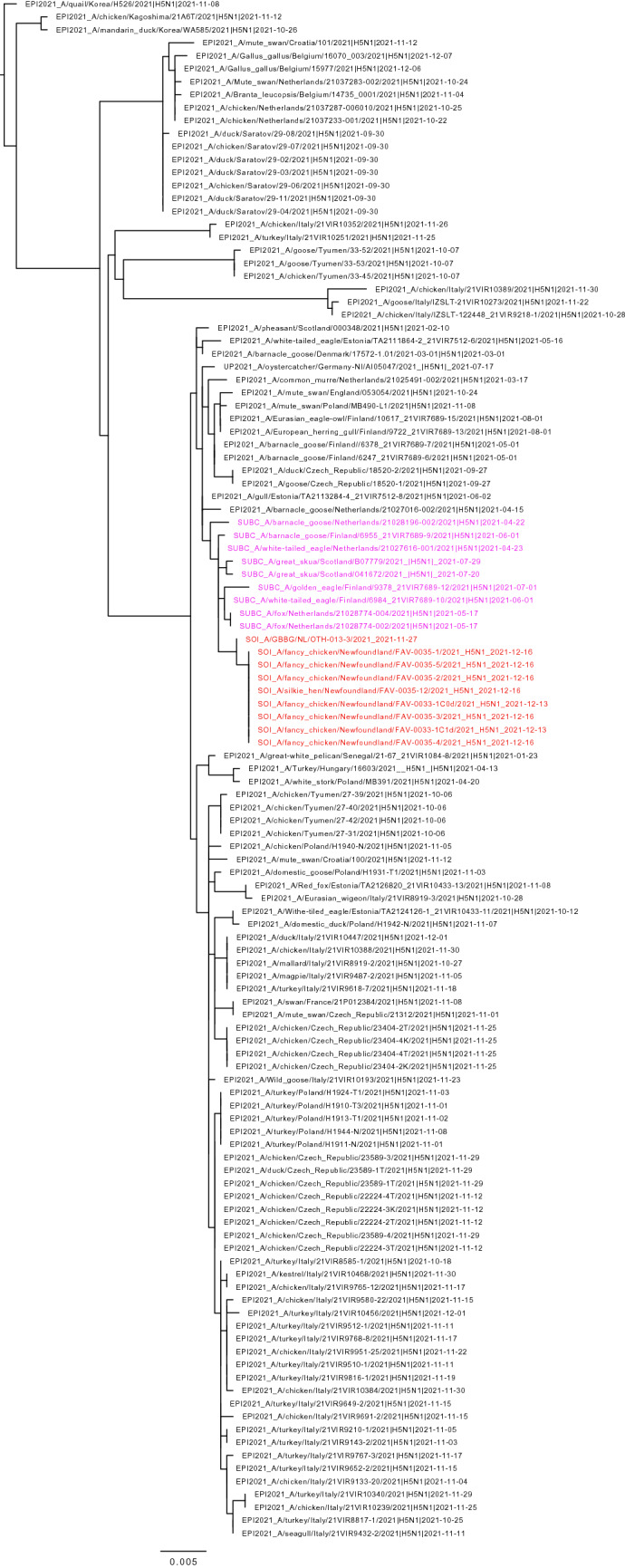

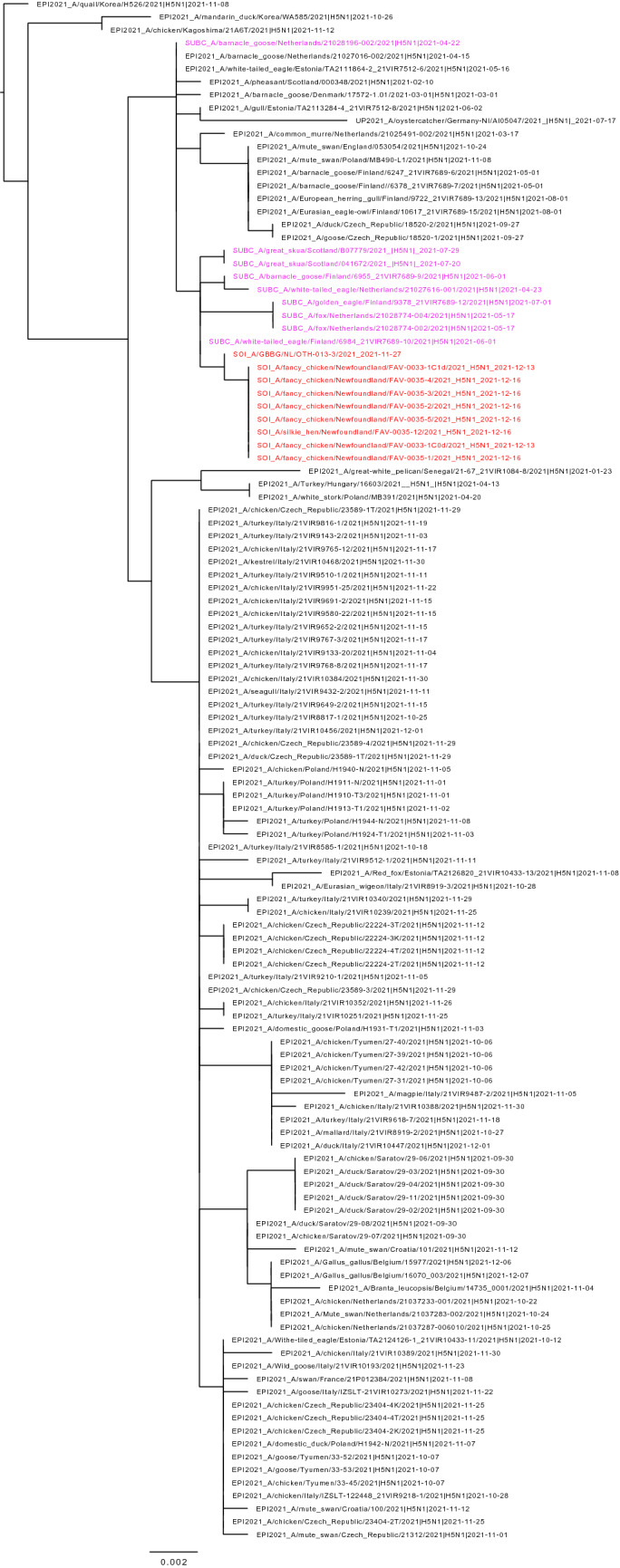

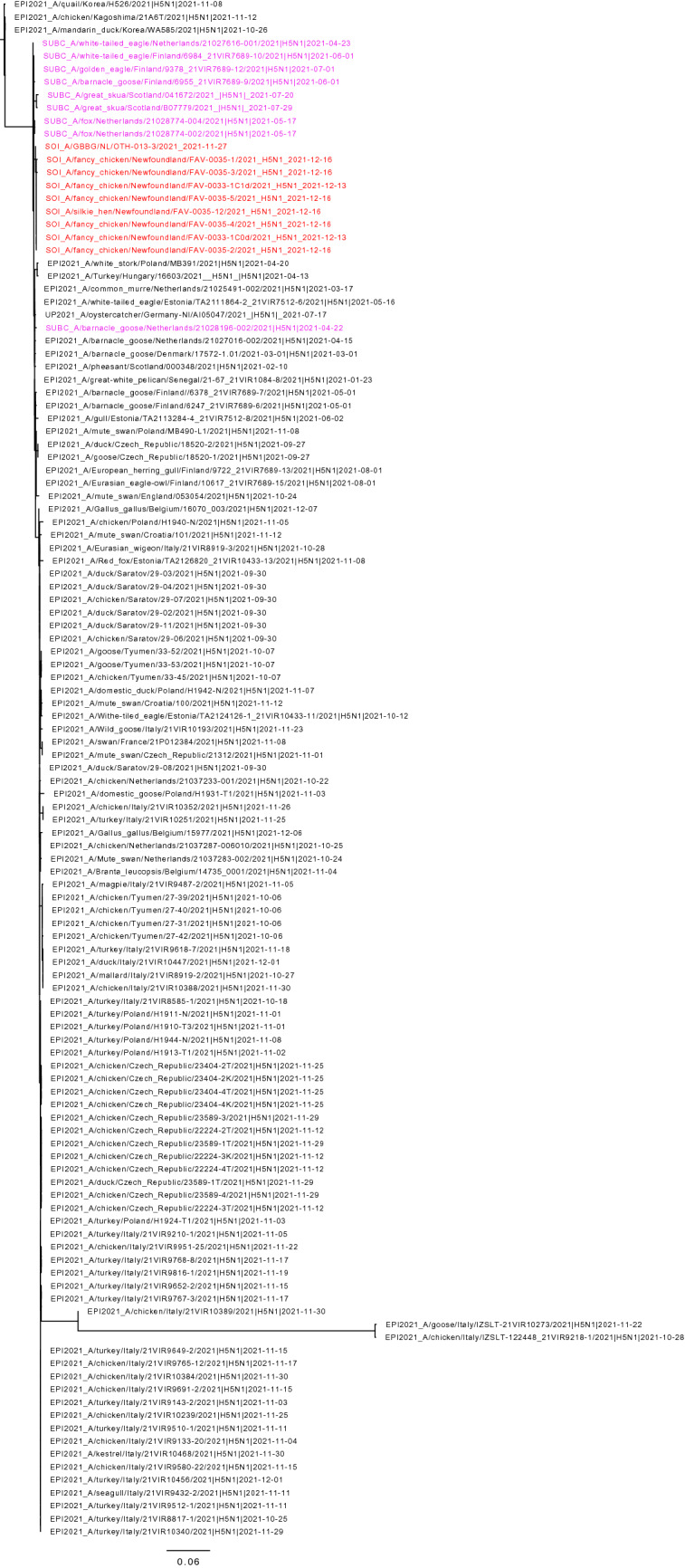
Maximum likelihood phylogenetic tree of the H5 gene segments. Relationships among the European 2021 H5 2.3.4.4b HPAI strains (magenta) and the Newfoundland wild bird and outbreak strains (red) are shown. The tree is rooted by the outgroup and nodes placed in descending order; order: HA, NA, PA, PB1, PB2, NP, MP, NS.

**Figure 3:**
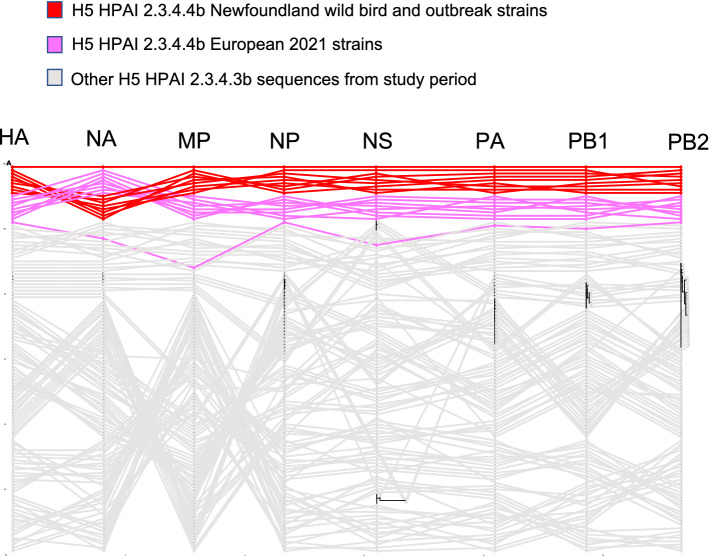
Phylogenetic incongruence analyses. Maximum likelihood trees for the H and N gene segments and internal gene segments from equivalent strains were connected across the trees. Tips and connecting lines are coloured according to the legend**.**

Phylogenetic analyses were performed to compare the genome sequences of the Newfoundland viruses from the exhibition farm birds and a great black-backed gull found nearby to other influenza viruses in the database. Based on BLAST analysis all eight gene segments of the virus had a Eurasian origin, and the virus was identified as a clade 2.3.4.4b H5N1 virus. Based on maximum likelihood and time-resolved trees inferred by use of whole genome sequences, the Newfoundland viruses had a shared common ancestor with European viruses circulating in early 2021 (Figs. 1, 2). The dates for the most recent common ancestor (MRCA) of all gene segments ranged from December 2019 to April 2021 (Table 3). There was no evidence that the Newfoundland viruses were genetically closely related to other current or recent viruses circulating in Europe. In contrast to currently circulating European viruses, the sequences of the Newfoundland viruses had no evidence of reassortment with other avian influenza viruses after ancestral emergence (Fig. 3). The virus from the great black-backed gull was highly similar to those from the exhibition farm, except for a small number of nucleotide differences in the neuraminidase (N) gene segment.

**Table 3:** Dates for the most recent common ancestor (MRCA) of all gene segments.

Gene segment	Node date	Lower bound	Higher bound
PB2	13 February 2021	6 March 2017	24 April 2021
PB1	4 January 2021	30 August 2020	17 April 2021
PA	22 February 2021	12 September 2020	24 April 2021
NS	18 January 2021	5 April 2020	2 June 2021
NP	31 August 2020	25 August 2018	23 April 2021
NA	5 April 2021	27 February 2021	24 April 2021
HA	20 August 2021	17 June 2021	19 October 2021
MP	8 August 2021	18 April 2021	29 November 2021

### Analysis of avian migration and potential routes for HPAI H5 virus to be carried across the Atlantic with migrating birds

**Figure 4:**
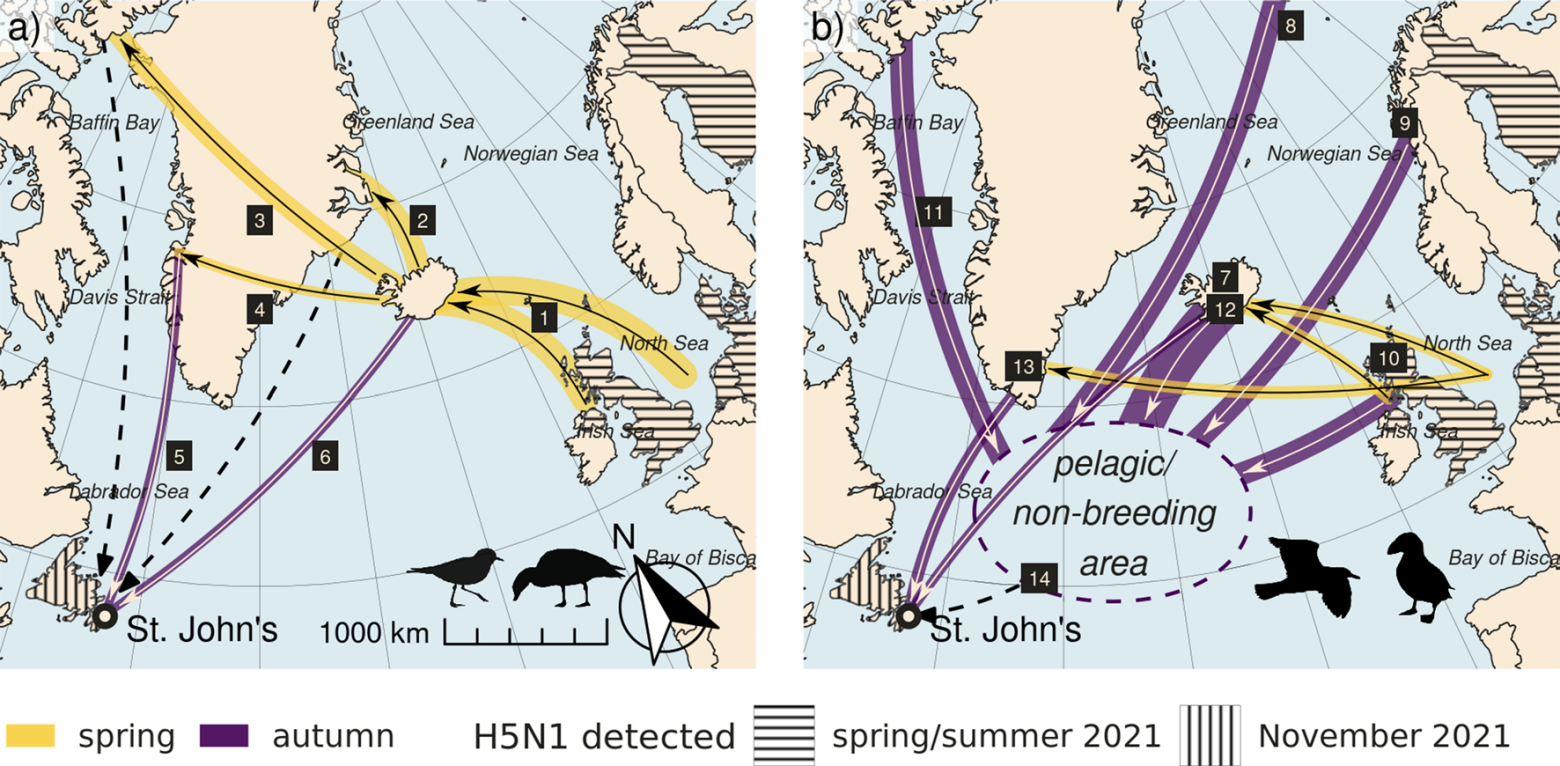
Maps of transatlantic migration. Putative virus transmission pathways between Europe and Newfoundland via migratory waterfowl/shorebirds (**a**) and pelagic seabirds (**b**). Many Icelandic waterfowl and shorebirds (**a**) winter in Northwest Europe and return to Iceland to breed in spring (1), including whooper swans, greylag geese, pink-footed geese, Eurasian wigeons, Eurasian teals, northern pintails, common ringed plovers and purple sandpipers. Some bird populations use Iceland as a stopover site, and continue to breeding grounds in East Greenland (2; barnacle geese and pink-footed geese), the East Canadian Arctic (3; light-bellied brent geese, red knots, ruddy turnstones) and West Greenland (4; greater white-fronted geese). Migratory birds from Europe share these breeding areas with species that winter in North America, including Canada geese and snow geese from East Greenland and the East Canadian Arctic (5), and some Iceland-breeding species of duck, including small numbers of Eurasian wigeons, Eurasian teals, and tufted ducks (6). Several seabird species (**b**), such as gulls, skuas, fulmars and auks, have large breeding ranges in the Arctic. After the breeding season many species become fully pelagic and can roam large parts of the northern Atlantic. The mid-Atlantic ridge outside Newfoundland is an important non-breeding area for seabirds, and is frequented by auks from Iceland (7), Svalbard (8) and Norway (9), including large numbers of Atlantic puffins and Brünnich guillemots, and by black-legged kittiwakes and northern fulmars originating from Iceland, Norway and the United Kingdom (7–8, 10). There these birds are joined by seabirds from Canadian and Greenlandic waters (11). Direct migratory links to Newfoundland occurs through greater and lesser-black backed gulls as well as black-headed gulls from Iceland and Greenland (12, 13), and gulls also link the pelagic and the coastal zone around Newfoundland (14). Thickness of the lines highlights the relative approximate population sizes. Dashed lines show where small numbers of individuals, or vagrants, provide a potential pathway. For more details on species and population numbers see Table 2. This figure was prepared using the software R (version 4.0.5, https://www.r-project.org/) and the following packages: -ggplot2 (version 3.3.5, https://cran.r-project.org/web/packages/ggplot2/index.html), -sf (version 1.0.5, https://cran.r-project.org/web/packages/sf/index.html).

There are several pathways for HPAI H5N1 virus to be carried across the Atlantic with migrating birds, based on the multitude of migration routes of wild birds and their overlapping ranges at breeding, stop-over, and wintering sites. Ring-recovery data confirm the regular movements of wild birds from Europe to Iceland and other North Atlantic islands, and from there to North America, and also provide evidence for direct movements of for example seabirds and gulls (Supplementary Table 1). Ringed individuals with a European origin have been found on Newfoundland for 10 of the 24 species in Supplementary Table 1: barnacle goose (1 ringed individual), Eurasian wigeon (5), Eurasian teal (1), great skua (13), European herring gull (1), black-headed gull (1), black-legged kittiwake (102), purple sandpiper (1), Brunnich's guillemot (15), and Atlantic puffin (50). Given that the most likely ancestor of the virus detected in Newfoundland was circulating in Northwest Europe between the beginning of the 2020/2021 outbreak in Europe in October 2020 and April 2021 (see above), likely routes include spring migration of bird species moving to Icelandic, Greenlandic or Canadian High Arctic breeding grounds, or migration directly across the Atlantic Ocean (Fig. 4).

The first possible route via Iceland seems to be the strongest link between Newfoundland and Europe^14–17^, because it is a meeting point of breeding bird populations which winter in Europe as well as along the East coast of North America. Numerous species, totaling almost two million individual birds, migrate annually from northwestern Europe to breeding grounds in Iceland and beyond. Several populations breed on Iceland, including swans (whooper swan) (Supplementary Table 1), geese (greylag goose, pink-footed goose), ducks (Eurasian wigeon, Eurasian teal, Northern pintail), gulls (great- and lesser black-backed gull, black-headed gull, black-legged kittiwake) and shorebirds (common ringed plover, purple sandpiper, Supplementary Table 1). In addition, several species (e.g. barnacle geese and pink-footed geese) migrating to breeding grounds further away (Greenland and/or Canadian High Arctic) make spring and autumn stopovers in Iceland^18,19^. This creates potential for the virus to have been spread northwards to Iceland (or further northward) in spring, where it could have circulated among breeding birds, or transmitted during autumn migration by species returning from the Arctic. Several Iceland-breeding species of ducks (Eurasian wigeon, Eurasian teal, tufted duck), gulls (lesser black-backed gull, black-legged kittiwake, black-headed gull) and alcids (Brunnich’s guillemot, Atlantic puffin) winter along the Atlantic coast of North America in variable numbers (Supplementary Table 1). If the virus was transmitted to one of these populations during their stay on Iceland, it could have been spread to Newfoundland during the subsequent autumn migration. Importantly, Iceland-breeding Eurasian wigeons or Eurasian teals could be responsible for both the journey to Iceland from European wintering grounds, as well as the journey from Iceland to Newfoundland, where these species are frequently encountered as vagrants (Supplementary Table 1)^20,21^.

The second possible route is via species that migrate from northwestern Europe to the Canadian High Arctic and/or Northwest Greenland. These include shorebirds (e.g. ruddy turnstone, red knot) and some geese (light-bellied brent goose and greater white-fronted goose). If the virus circulated in these breeding populations and then moved to other coastal marine bird populations bordering Baffin Bay, which include huge numbers of colonial seabirds and marine waterfowl^22,23^, the virus could have followed a coastal or even pelagic route south with the large autumn migration of Arctic marine birds (various sea ducks, auks and larids)^24,25^ to emerge in Newfoundland. Alternatively, shorebirds and waterfowl may have played a role: several European-wintering populations have overlapping breeding grounds with populations wintering along the east coast of North America. Regarding geese, greater white-fronted geese share breeding grounds in western Greenland with Canada geese^26,27^, which migrate south along the Canadian Atlantic coast. Also, brent geese have overlapping breeding grounds with snow geese^18^. In addition, individual barnacle geese and pink-footed geese breeding in Greenland could also have travelled south to Newfoundland carrying the virus, as these birds are regular vagrants to the North American Atlantic coast^28^. While geese occur only in small numbers on Newfoundland, two barnacle geese and four pink-footed geese, probably originating from Greenland breeding grounds, were observed in the autumn of 2021. St. John’s is the first major population center on a coastal route south from Baffin Bay/Davis Strait and along the Labrador Shelf, so emergence in eastern Newfoundland is consistent with this route.

Three wild bird species involved in the Iceland and/or Greenland/High Canadian Arctic routes deserve particular attention. Eurasian wigeon have been prominently involved in outbreaks in Eurasia, and are considered prime candidates for carrying HPAI virus over long distances^29^. Also, during the first stages of an outbreak they are one of the first species to be detected HPAI virus positive, often without clinical signs. Barnacle geese and greylag geese, which congregate in Iceland, were in the top three most abundant species detected H5-positive in Europe in late winter and early spring 2021^5^. Given that both greylag and barnacle geese have populations breeding on Iceland/Greenland and wintering in Europe (particularly the UK), these two species are high on the list of probable vectors that transported the virus to Iceland/Greenland and finally to Newfoundland. The high involvement of infected geese in the HPAI dynamics, which was not seen before October 2020, together with the unusually high levels of HPAI H5 virus presence in wild birds in Northwest Europe in spring 2021, might also explain why HPAI H5 virus spread to Newfoundland this winter (2021/2022), and not in the previous winters (2020/2021, 2016/2017, 2014/2015, 2005/2006). It is, however, striking that no cases of HPAI H5 virus have been recorded on Iceland in 2021.

A third possible, pelagic, route is directly across the Atlantic Ocean. Such a route could have started with coastal and pelagic seabirds in Northwest Europe, where the virus may have remained undetected for much of the summer of 2021. A subsequent migration of seabirds to Newfoundland waters in the autumn of 2021 could have brought the virus to North America. The large populations of black-legged kittiwakes and northern fulmars that breed in the U.K. have long been known to frequent Newfoundland waters^30^, and these movements have been corroborated by recent telemetry studies^31^. Further, recent telemetry information reveals that millions of pelagic seabirds breeding all across the Atlantic congregate over the Mid-Atlantic Ridge in the central North Atlantic at all times of year^32^, making a pelagic transmission route a possibility. From the pelagic wintering grounds off Newfoundland, a species that uses both pelagic and coastal habitats, possibly a gull, may have brought the virus to shore in St. John’s. Trans-Atlantic transmission via seabirds has been suggested for LPAI viruses, including detection of mosaic Eurasian-North American viruses in gulls and alcids^12,33–35^.

For the time period and geographical frame considered**,** HPAI-H5-positive species included ducks (Eurasian wigeon, mallard, common eider), geese (barnacle, greylag, brent, pink-footed and greater white-fronted goose), swans (whooper), gulls (black-headed, herring, lesser black-backed, great black-backed), and shorebirds (red knot, ruddy turnstone) (Supplementary Table 2). Of these 15 species, ringed individuals with a European origin have been recorded on Newfoundland for barnacle goose (1 ringed individual), Eurasian wigeon (5), great skua (13), and black-headed gull (1) (Supplementary Table 1). Ringed individuals with a European origin have been found on Newfoundland for 5 species which were found to be HPAI-H5-positive between October 2020 and April 2021, such as Barnacle Goose (1), Eurasian Wigeon (5), Great Skua (13), Black-headed Gull (1). These species might be considered to be possible carriers of HPAI H5 virus from Europe in late winter 2020/2021 or early spring 2021 partly or all the way to Newfoundland. However, given the incompleteness of sampling and the possibility of wild birds carrying HPAI virus subclinically, the involvement of other wild bird species in transatlantic virus transport cannot be ruled out.

Having reached the Avalon Peninsula of Newfoundland via one of above routes, the virus may have spread further within the abundant local populations of ducks and gulls wintering in the city of St. John’s. The peridomestic populations of some of these species may be candidates for incursion of the virus into the farm in St John’s.

## Conclusions

In conclusion, the HPAI H5N1 viruses that were detected in Newfoundland in November and December 2021 originated from Northwest Europe and belonged to HPAI clade 2.3.4.4b. Most likely, these viruses emerged in Northwest Europe in winter 2020/2021, dispersed from Europe in late winter or early spring 2021, and arrived in Newfoundland in autumn 2021. The viruses may have been carried across the Atlantic by migratory birds using different routes, including Icelandic, Greenland/Arctic, or pelagic routes. The unusually high presence of the viruses in European wild bird populations in late winter and spring 2021, as well as the greater involvement of barnacle and greylag geese in the epidemiology of HPAI in Europe since October 2020, may explain why spread to Newfoundland happened this winter (2021/2022), and not in the previous winters.

The incursion of these HPAI viruses, which appear to be well-adapted to certain wild birds, raised concern at its first detection about the potential of HPAI virus to become established and spread in the Americas via wild birds (OFFLU reference). The concern was that if these viruses become established in the Atlantic Flyway, they could rapidly spread west to Mississippi, Central and Pacific Flyways. The implication of this scenario would be high wild bird mortality, risk for incursion into poultry holdings and those of other captive birds, as well as zoonotic risk. In fact, as of 4 April 2022, this virus has now been detected in wild birds in three flyways in North America and disease in commercial and/or backyard poultry flocks has been detected in 3 Canadian provinces and 24 US states (https://www.usgs.gov/centers/nwhc/science/distribution-highly-pathogenic-avian-influenza-north-america-20212022). Large scale mortality events have not been reported in wild birds, with most positive wild birds detected via testing of hunter-collected birds or testing of individual dead birds (https://www.aphis.usda.gov/aphis/ourfocus/animalhealth/animal-disease-information/avian/avian-influenza/hpai-2022/2022-hpai-wild-birds).

To prevent and mitigate the risk of viral spread, it will be vital to further increase surveillance of wild birds in North America and South America, as well as at migration stop-over stations in Iceland and Greenland. This should include virus detection with whole genome sequencing to enable molecular epidemiology. Collecting wild bird mortality reports can give an idea of the impact of the outbreak on local wild bird populations, and active surveillance is critical to identify vector species. The overlap of migratory movements of wild waterbirds along the Atlantic coast of North America with densely populated poultry areas may increase the risk of viral incursion into poultry farms, emphasizing the need for appropriate biosecurity measures and spatial planning of the poultry sector. The spread of HPAI H5 viruses from Europe to North America stresses the importance of close international cooperation and data exchange to better understand the global epidemiology of avian influenza, e.g. by swiftly identifying where these viruses emerged from and mitigating endemic disease in poultry to avoid these production systems potentially acting as future sources for emerging variants^35^ , and is a call to re-assess the poultry sector in a way that embraces the One Health perspective: to sustainably balance and optimize the health of people, animals and ecosystems (https://www.who.int/groups/one-health-high-level-expert-panel).

## Materials and methods

### Phylogenetic analysis

Methods for phylogenetic analysis were the same as Sagulenko 2018 and Poen 2019^36,37^.

Full genome sequences were obtained from nine clinical or postmortem samples of captive birds at the exhibition farm, and from one postmortem sample of a great black-backed gull from a nearby city pond.

We searched for H5NX whole genome sequences in GISAID from Europe, Asia and Africa where samples were collected from 01-01-2021 through 27-12-2021. To these existing data we added eight unpublished sequences from Newfoundland, and three additional unpublished sequences from European wild birds collected in the timeframe to the GISAID database.

We aligned the sequences using MAFFT v7.407 and trimmed to the starting ATG and ending STOP codon. Maximum-likelihood trees were inferred using IQ-TREE 2.1.3. and 1000 replicates for the Shimodaira–Hasegawa approximate likelihood ratio test. We used TreeTime, a Python-based framework for phylodynamic analysis using an approximate Maximum Likelihood approach to estimate ancestral states, and reroot trees to maximize temporal signals.

### Analysis of avian migration

We evaluated the possible routes along which wild birds can migrate from Europe to North America, based on knowledge on existing migration routes as well as the retrieval of identification (bird) rings. We compared the information with the data of HPAI H5 virus-positive birds from Northwest European countries (i.e., UK, Ireland, Norway, Finland, Denmark, Germany, Netherlands, Belgium, France) that are the starting points, or are situated along these migratory routes. For the analysis, we prioritized the most abundant bird species, that also most frequently tested H5-positive during the 2020/2021 outbreak in Europe, as detected during active and passive surveillance^38^.

We focused on bird species susceptible to avian influenza (waterfowl, gulls, shorebirds and seabirds) which either bred or made a migratory stopover on Iceland, this being the most likely connection between Europe and Newfoundland. We identified wintering grounds, staging sites and breeding grounds based on literature, using mostly the database of Birds of the World^39,40^. We estimated the population sizes breeding in Iceland or passing through Iceland during migration using Fox & Leafloor (2018), Icelandic Institute of Natural History (2021) and van Roomen (2018)^40–42^.

We provided the number of individuals observed in Newfoundland from Ebird data^19^. We extracted all observations from complete lists done between September–December, 2011 to 2021 on Newfoundland. For rare species (with less than 10 records annually) we also included sightings from incomplete lists. For every year and species, we calculated the maximum number of observed individuals per location, and added these to calculate the total number of individuals observed in Newfoundland for every year. We then calculated the average number of individuals observed annually between 2011 and 2021, and the number observed in 2021. We identified the most likely origin of birds encountered in Newfoundland using the database Birds of the World^39–43^.

Ring-recovery data were obtained from the EURING Migration mapping Tool MMT, an online tool under development, that provides information on movements of ringed birds between pre-set areas within Europe and to other areas of the world, based on the EURING databank. These data were augmented with published (individuals recovered up to 2002, Lyngs 2003)^12–16^, and unpublished data (to 2011) of birds ringed in Greenland supplied by Copenhagen Bird Ringing Centre. All records of individual birds moving between Northwest Europe (Norway & Sweden, Germany & Denmark, Belgium & Netherlands, Great Britain & Ireland) and Iceland and Faroe Islands, or Svalbard and other North Atlantic islands or Greenland, and individual birds moving between these areas and Canada or USA were selected. Prior to selection, unlikely records (finding date before ringing date, finding or ringing location not accurate etc.) were removed. For species not considered in the Migration Mapping Tool, only records of birds moving between Northwest Europe and Greenland, Canada or USA were available.

To further evaluate which wild bird species might have been involved in transatlantic transport of HPAI H5 virus, we compared above bird migration patterns with reports of HPAI-H5-positive wild birds in Europe. We limited our evaluation to the period of six months up to April 2021, the latest MRCA date of the Newfoundland virus gene segments (see above), and to the coastal countries in Northwest Europe, which act as the main wintering areas for wild birds that migrate across the Atlantic.

## Supplementary Information

Supplementary Table 1.

Supplementary Table 2.

## Data Availability

All data are available in the main text or the supplementary materials. To request the data from this study please contact the corresponding author. Email: t.kuiken@erasmusmc.nl.

## References

[1] Duan L (2008). The development and genetic diversity of H5N1 influenza virus in China, 1996–2006. Virology.

[2] Global Consortium for H5N8 and Related Influenza Viruses. Role for migratory wild birds in the global spread of avian influenza H5N8. *Science* **354**, 213–217. 10.1126/science.aaf8852 (2016). 10.1126/science.aaf8852 PMC5972003 27738169

[3] Lycett SJ (2020). Genesis and spread of multiple reassortants during the 2016/2017 H5 avian influenza epidemic in Eurasia. Proc. Natl. Acad. Sci. U S A..

[4] Banyard AC (2022). Detection of highly pathogenic avian influenza virus H5N1 clade 2.3.4.4b in Great Skuas: A species of conservation concern in Great Britain. Viruses.

[5] EFSA (European Food Safety Authority) *et**al*. *EFSA**Scientific**Report:**Avian**Influenza**Overview**September–December**2021*. 1–94. 10.2903/j.efsa.2021.7108 (2021). 10.2903/j.efsa.2021.7108 PMC8698678 34987626

[6] King J (2021). The genetics of highly pathogenic avian influenza viruses of subtype H5 in Germany, 2006–2020. Transbound. Emerg. Dis..

[7] Zecchin B (2021). Evolutionary dynamics of H5 highly pathogenic avian influenza viruses (Clade 2.3.4.4B) circulating in Bulgaria in 2019–2021. Viruses.

[8] Olsen B (2006). Global patterns of influenza a virus in wild birds. Science.

[9] Adlhoch C, Baldinelli F, Fusaro A, Terregino A (2021). Avian influenza, a new threat to public health in Europe?. Clin. Microbiol. Infect..

[10] Xu W (2016). Epidemiological and evolutionary inference of the transmission network of the 2014 highly pathogenic avian influenza H5N2 outbreak in British Columbia, Canada. Sci. Rep..

[11] OIE 2021, report id. CAN-2021-HPAI-001. https://wahis.oie.int/#/report-info?reportId=45054. Accessed 08 Jan 2022 (2022).

[12] Dusek RJ (2014). North Atlantic migratory bird flyways provide routes for intercontinental movement of avian influenza viruses. PLoS ONE.

[13] Huang Y (2014). Genetic structure of avian influenza viruses from ducks of the Atlantic flyway of North America. PLoS ONE.

[14] Canadian Wildlife Service Waterfowl Committee. *Population**Status**of**Migratory**Game**Birds**in**Canada.**November**2019.**CWS**Migratory**Birds**Regulatory**Report**Number**52* (2020).

[15] Fifield, D.A., Lewis, K.P., Gjerdrum, C., Robertson, G.J. & Wells, R. *Offshore**Seabird**Monitoring**Program.**Environment**Studies**Research**Funds**Report**No.**183.**St.**John’s*. 1–68. (2009).

[16] Lyngs P (2003). Migration and winter ranges of birds in Greenland. Dansk Ornitol. Forenings Tidsskrift..

[17] Hall JS (2014). Avian influenza virus ecology in Iceland shorebirds: Intercontinental reassortment and movement. Infect. Genet. Evol..

[18] Fox, A.D. & Leafloor J.O. (eds.) *A**Global**Audit**of**the**Status**and**Trends**of**Arctic**and**Northern**Hemisphere**Goose**Populations**(Component**2:**Population**accounts).**Conservation**of**Arctic**Flora**and**Fauna**International**Secretariat:**Akureyri,**Iceland.* ISBN 978-9935-431-74-5 (2018).

[19] eBird. 2022. *eBird:**An**Online**Database**of**Bird**Distribution**and**Abundance**[Web**Application]*. http://www.ebird.org. Accessed 4 Jan 2022 (eBird, Cornell Lab of Ornithology, 2022).

[20] Howell SNG, Lewington I, Russell W (2014). Rare Birds of North America.

[21] Edgell MC (1984). Trans-hemispheric movements of Holarctic Anatidae: The Eurasian wigeon (*Anas*
*penelope* L.) in North America. J. Biogeogr..

[22] Mallory, M.L. & Fontaine, L.A.J. (eds.) Key marine habitat sites for migratory birds in Nunavut and the Northwest Territories. in *Canadian**Wildlife**Series**Occasional**Paper**Number**109*. (Environment Canada, 2004).

[23] Gaston AJ, Mallory ML, Gilchrist HG (2012). Population and trends of Canadian Arctic seabirds. Polar Res..

[24] Fifield DA (2017). Employing predictive spatial models to inform conservation planning for seabirds in the Labrador Sea. Front. Mar. Sci..

[25] Wong SNP, Gjerdrum C, Morgan KH, Mallory ML (2014). Hotspots in cold seas: The composition, distribution, and abundance of marine birds in the North American Arctic. J. Geophys. Res. Oceans..

[26] Kuletz, K. *et**al*. Seabirds. in *State**of**the**Arctic**Marine**Biodiversity**Report.**Conservation**of**Arctic**Flora**and**Fauna*. 139–165. (Circumpolar Biodiversity Monitoring Plan, Arctic Council, 2017).

[27] Fox AD (1996). North American Canada Geese (*Branta*
*canadensis*) in West Greenland. Auk.

[28] Sherony DF (2008). Greenland geese in North America. Birding.

[29] van den Brand J (2018). Wild ducks excrete highly pathogenic avian influenza virus H5N8 (2014–2015) without clinical or pathological evidence of disease. Emerg. Microbes Infect..

[30] Tuck LM (1971). The occurrence of Greenland and European birds in Newfoundland. Bird-Banding.

[31] Frederiksen M (2012). Multicolony tracking reveals the winter distribution of a pelagic seabird on an ocean basin scale. Divers. Distrib..

[32] Davies TE (2021). Multispecies tracking reveals a major seabird hotspot in the North Atlantic. Conserv. Lett..

[33] Wille M, Robertson GJ, Whitney H, Ojkic D, Lang AS (2011). Reassortment of American and Eurasian genes in an influenza A virus isolated from a great black-backed gull (*Larus*
*marinus*), a species demonstrated to move between these regions. Arch. Virol..

[34] Lang AS (2016). Assessing the role of seabirds in the ecology of influenza A viruses. Avian Dis..

[35] Lewis NS (2021). Emergence and spread of novel H5N8, H5N5 and H5N1 clade 2.3.4.4 highly pathogenic avian influenza in 2020. Emerg. Microbes Infect..

[36] Sagulenko P, Puller V, Neher RA (2018). TreeTime: Maximum-likelihood phylodynamic analysis. Virus Evol..

[37] Poen MJ (2019). Co-circulation of genetically distinct highly pathogenic avian influenza A clade 2.3.4.4 (H5N6) viruses in wild waterfowl and poultry in Europe and East Asia, 2017–18. Virus Evol..

[38] Gobbo F, Fornasiero D, De Marco MA, Zecchin B, Mulatti P, Delogu M, Terregino C (2021). Active surveillance for highly pathogenic avian influenza viruses in wintering waterbirds in northeast Italy, 2020–2021. Microorganisms.

[39] Billerman, S.M., Keeney, B.K., Rodewald, P.J. & Schulenberg, T.S. (eds). *Birds**of**the**World*. https://birdsoftheworld.org/bow/home. (Cornell Laboratory of Ornithology, 2020)

[40] Fox, A.D & Leafloor, J.O. (eds.). *A**Global**Audit**of**the**Status**and**Trends**of**Arctic**and**Northern**Hemisphere**Goose**Populations**(Component**2:**Population**Accounts).* ISBN 978-9935-431-74-5 (Conservation of Arctic Flora and Fauna International Secretariat, 2018).

[41] Icelandic Institute of Natural History. https://en.ni.is/biota/animalia/chordata/aves. Accessed 30 Dec 2021 (2021).

[42] van Roomen, M. *et**al*. East Atlantic Flyway. in *Wadden**Sea**Quality**Status**Report**2017*. (eds. Kloepper, S. *et**al*.) (Common Wadden Sea Secretariat, 2018).

[43] Brides K (2021). The Icelandic Whooper Swan *Cygnus*
*cygnus* population: Current status and long-term (1986–2020) trends in its numbers and distribution. Wildfowl.

